# Reaction Mechanism of ZrB_2_-ZrC Formation in Ni-Zr-B_4_C System Analyzed by Differential Scanning Calorimetry

**DOI:** 10.3390/ma14216467

**Published:** 2021-10-28

**Authors:** Jiaying Xu, Pengfei Ma, Binglin Zou

**Affiliations:** 1 Jilin Institute of Chemical Technology, College of Science, Jilin 132022, China; mapf@jlict.edu.cn; 2 State Key Laboratory of Rare Earth Resources Utilization, Changchun Institute of Applied Chemistry, Chinese Academy of Sciences, Changchun 130022, China; zoubinglin@ciac.ac.cn

**Keywords:** reaction mechanism, ZrB_2_-ZrC, combustion synthesis, self-propagation high-temperature synthesis, differential scanning calorimetry

## Abstract

The reaction mechanism of ZrB_2_-ZrC formation in a 30% Ni-Zr-B_4_C system under argon was revealed by using differential scanning calorimetry (DSC), X-ray diffraction (XRD) and scanning electron microscopy (SEM). The results indicated that the reaction mechanism in the Ni-Zr-B_4_C system was complex. Initially, Ni_x_Zr_y_ and Ni_x_B_y_ intermetallics were formed via solid-state diffusion reactions between Ni, B_4_C and Zr. Then, the eutectic reaction between Ni_2_B and Ni_4_B_3_ lead to the formation of Ni-B liquid. The free C atoms dissolved into the Ni-B liquid to form a Ni-B-C ternary liquid, and then part of the Zr powder dissolved into the surrounding Ni-B-C ternary liquid to form Ni-Zr-B-C quaternary liquid. Finally, ZrB_2_ and ZrC formed and precipitated out of the saturated liquid. The eutectic liquid plays an important role during the formation of ZrB_2_-ZrC.

## 1. Introduction

Boride and carbide of zirconium (ZrB_2_ and ZrC) exhibit outstanding properties such as high hardness and melting points, low density as well as high resistance to corrosion and wear, which makes them attractive candidates for high-temperature ceramics, cutting tools, corrosion-resistant parts, reinforcing particles in the composites and wear resistant coatings [1,2,3,4,5,6]. It is believed that double or multiple phase ceramics have better properties than single-phase ceramics [7,8,9]. Hence, more attention has been paid to develop materials combining ZrB_2_ and ZrC ceramics [1,2,3,4,5].

Multiphase ceramics can be synthesized by a variety of methods including hot isostatic pressing, spark plasma sintering, pressureless sintering, combustion synthesis, etc. [10,11,12,13]. Among them, combustion synthesis (CS) has attracted much attention for preparation of intermetallics, borides, carbides, nitrides and silicides due to the advantages of simple devices, low processing cost, high reaction purity and fast reaction rates [14,15,16,17,18]. CS is generally divided into two types. The first is self-propagating high-temperature synthesis (SHS) by ignition and spread at one end of the sample, and the second is thermal explosion (TE) by heating the whole sample uniformly [19,20]. Up to now, ZrB_2_-ZrC/Al, ZrB_2_-ZrC/Cu and ZrB_2_-ZrC/Co composites have been successfully prepared by the SHS method [1,2,3,4,5].

In a previous paper [21], we successfully synthesized ZrC-ZrB_2_/Ni cermet powders using a Ni-Zr-B_4_C system by the SHS method. The SHS-derived feedstock powders were deposited on a magnesium alloy, and atmospheric plasma spraying was used to obtain ZrC-ZrB_2_/Ni cermet coatings. However, the reaction mechanism of ZrB_2_-ZrC formation in the Ni-Zr-B_4_C system needs to be further studied. 

In the present work, differential scanning calorimetry (DSC), X-ray diffraction (XRD) and scanning electron microscopy (SEM) were used to reveal the formation mechanism of ZrB_2_-ZrC in the Ni-Zr-B_4_C system during combustion synthesis. It is expected that these preliminary results will be valuable for promoting the understanding of the reaction mechanism of ZrB_2_-ZrC formation in the Ni-Zr-B_4_C system.

## 2. Materials and Methods

The ZrB_2_–ZrC/Ni composites were produced according to the following reaction equation:

(1) $$x{\mathrm{Ni}+3\mathrm{Zr}+B}_{4}C\to x\mathrm{Ni}+2{\mathrm{ZrB}}_{2}+\mathrm{ZrC}$$

Commercial powders Ni (~99% in purity, ≤48 μm, ST-nano science and technology Ltd. Co., Shanghai, China), Zr (~99% in purity, ≤38 μm, ST-nano science and technology Ltd. Co., Shanghai, China) and B_4_C (~95% in purity, ≤3.5 μm, Abrasive Ltd. Co., Dunhua, China) were selected as the starting materials. In order to investigate the complex combustion reactions in the Ni-Zr-B_4_C system, DSC experiments were performed on the mixtures of Zr-B_4_C, Ni-B_4_C, Ni-Zr and 30 wt.% Ni-Zr-B_4_C. In 30 wt.% Ni-Zr-B_4_C mixture, Zr and B_4_C powders with a molar ratio of 3:1 were mixed with 30 wt.% Ni. The compositional proportions in the Zr-B_4_C, Ni-B_4_C and Ni-Zr mixtures were in accordance with those in the 30 wt.% Ni-Zr-B_4_C mixture. The weight of powder mixtures subjected to DSC analysis was 15 mg. The reactant mixtures were dry-mixed sufficiently in a container using zirconia balls at a low speed (~50 rpm) for 6 h.

DSC was carried out on a STA 449C Jupiter (Netzsch, Weimar, Germany) apparatus to reveal the reaction mechanism of the Ni-Zr-B_4_C system. The heating process was set to a rate of 10 °C/min in flowing argon gas (99.9% in purity, flow rate: 40 mL/min). Following DSC analysis, the sintered powders were crushed, and the phase composition was analyzed by XRD (D8 Advance, Bruker, Ettlingen, Germany, Cu-Kα radiation, λ = 0.15406 nm) at a scanning speed of 6°/min and a scanning range of 20–80°. Microstructures of the reacted samples were characterized by SEM (S-4800, Hitachi, Tokyo, Japan) equipped with an energy-dispersive spectrometer (EDS).

## 3. Results and Discussion

**Figure 1:**
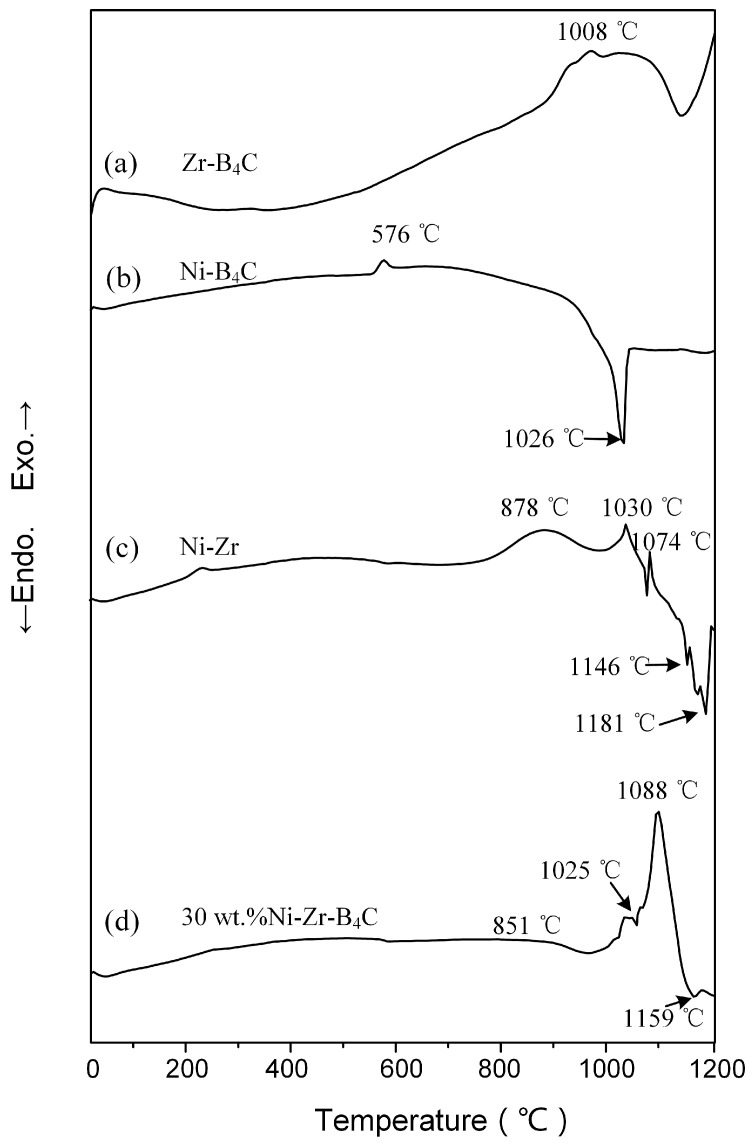
The DSC curves of the mixtures heated to 1200 °C with a heating rate of 10 °C/min: (**a**) Zr-B_4_C; (**b**) Ni-B_4_C; (**c**) Ni-Zr and (**d**) 30 wt.% Ni-Zr-B_4_C.

Figure 1 displays the DSC curves of the Zr-B_4_C, Ni-B_4_C, Ni-Zr and 30 wt.% Ni-Zr-B_4_C mixtures heated to 1200 °C with a heating rate of 10 °C/min. Moreover, interrupted experiments were performed in order to elucidate the reaction mechanism during the heating process.

**Figure 2:**
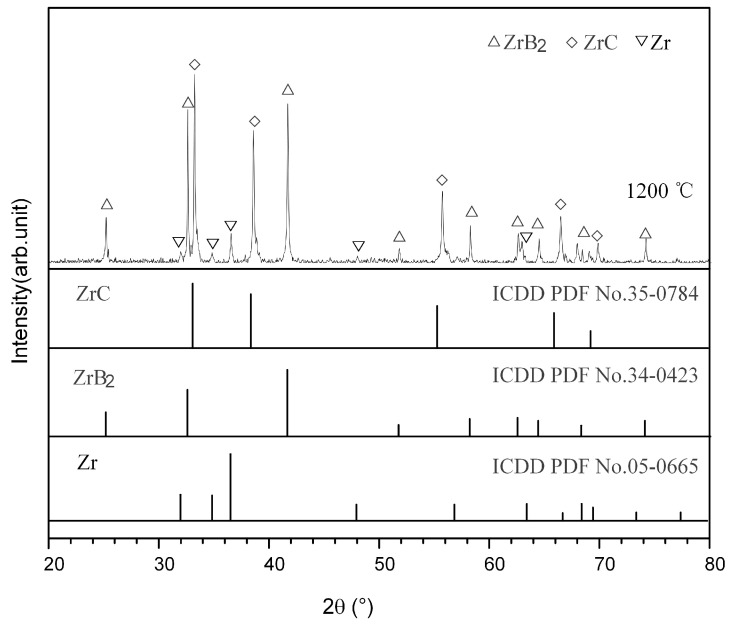
XRD pattern for the DSC product of the Zr-B_4_C mixture heated to 1200 °C.

The DSC curve of the Zr-B_4_C mixture is shown in Figure 1a. A broad exothermic peak appears near 1008 °C. The XRD result of DSC product heated to 1200 °C shows that the product mainly consists of a large amount of ZrB_2_, ZrC and a small amount of Zr (see Figure 2). The presence of Zr may have been caused by the incomplete reaction of reactants. Hu et al. [1] studied the mechanism of ZrB_2_ and ZrC generation in the Zr-B_4_C system and proposed that the solid-phase synthesis reaction was the main formation mechanism. Zhang et al. [2,3,4] investigated the reaction behavior and formation mechanism in the Cu-Zr-B_4_C system. Effects of heating rate and B_4_C particle size on the reaction process in the Zr-B_4_C system were also explored. Either increasing the particle size of B_4_C or increasing the heating rate may result in a sluggish solid-state reaction between Zr and B_4_C, which leads to the residual of Zr and B_4_C in the DSC products. The diffraction peaks of Zr were also found in the XRD patterns of the above research, but the diffraction peaks of B_4_C were very weak or absent due to the atomic characteristics and crystalline lattice of B_4_C [1,2,3,4].

**Figure 3:**
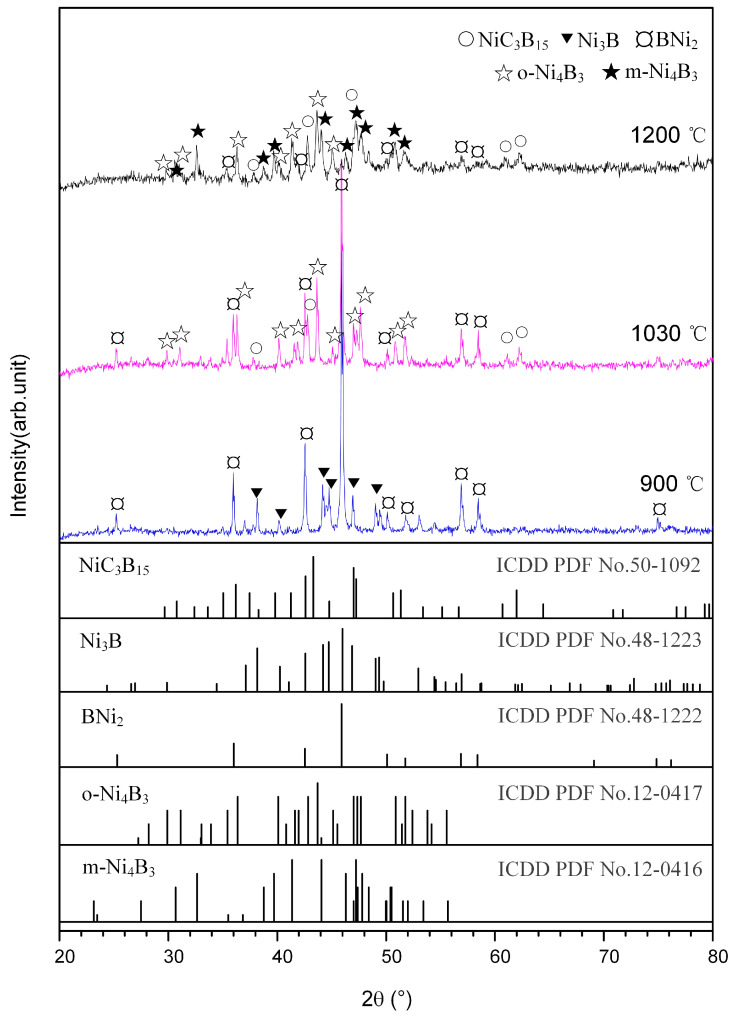
XRD patterns for the DSC products of Ni-B_4_C mixtures quenched at different temperatures.

Figure 1b shows the DSC curve of the Ni-B_4_C mixture heated to 1200 °C. A small exothermic peak was present at 576 °C, and a large endothermic peak was present at 1026 °C. To better interpret the two peaks, the Ni-B_4_C mixtures were heated to 900 °C and 1030 °C, respectively, before being cooled down. Figure 3 shows the XRD patterns obtained for DSC products when quenched from 900 °C, 1030 °C and 1200 °C, respectively. When the DSC heating was quenched from 900 °C, the product was mainly composed of Ni_2_B, Ni_3_B and C, indicating that the solid reaction between Ni and B_4_C occurred at this time, corresponding to the exothermic peak appearing at 576 °C on the DSC curve. As shown in Figure 3, the DSC product quenched from 1030 °C was mainly composed of Ni_2_B, o-Ni_4_B_3_ and a small amount of NiC_3_B_15_. When the Ni-B_4_C mixture was heated to 1200 °C, o-Ni_4_B_3_, m-Ni_4_B_3_ and a small amount of Ni_2_B and NiC_3_B_15_ were formed in the product. Following the Ni-B binary phase diagram [22], a Ni-B melt could be formed due to the eutectic reaction between Ni_2_B and o-Ni_4_B_3_ at 1018 °C, which corresponded to the large endothermic peak at 1026 °C on the DSC curve. At the same time, the Ni-B liquid phase could promote the dissolution of C atoms and form the Ni-B-C melt. The NiC_3_B_15_ phase was possibly formed and precipitated from it during the cooling process.

**Figure 4:**
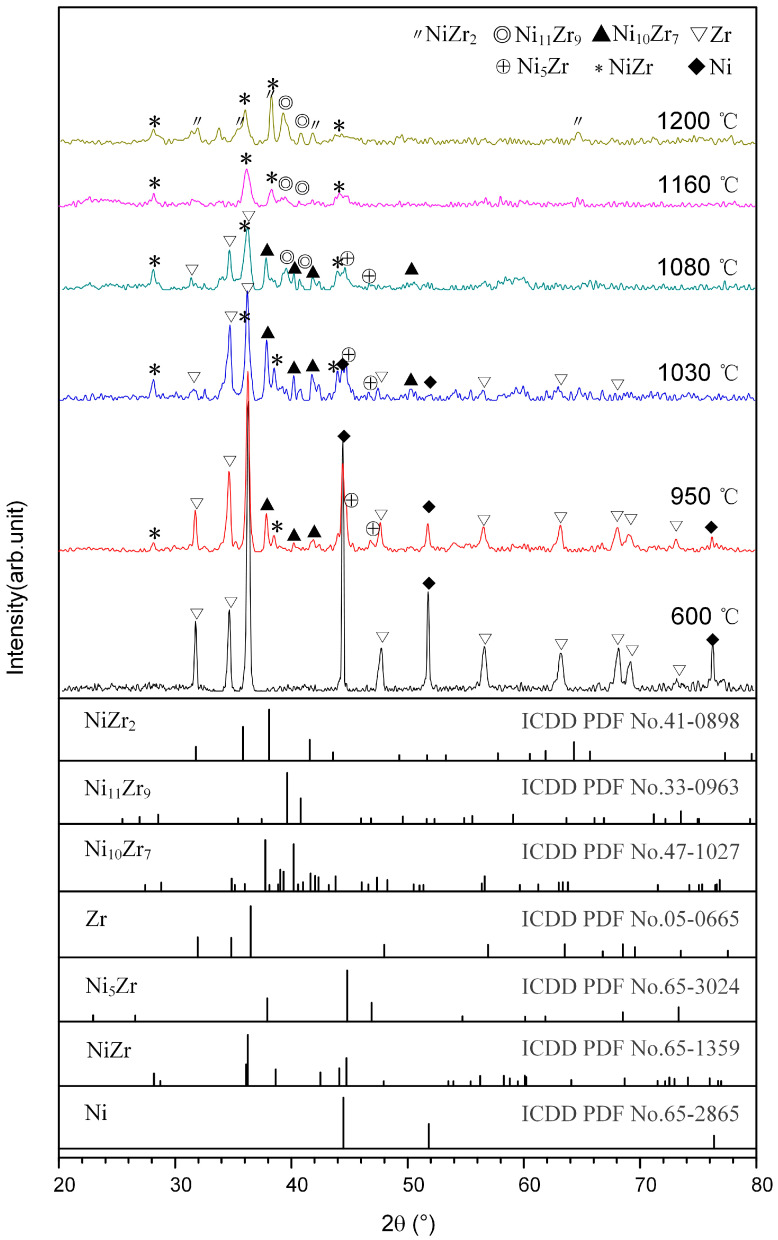
XRD patterns for the DSC products of Ni-Zr mixtures quenched at different temperatures.

Figure 1c shows the DSC curve of the Ni-Zr mixture heated to 1200 °C. As indicated, three exothermic peaks appear at 878 °C, 1030 °C and 1074 °C, respectively. Two endothermic peaks appear at 1146 °C and 1181 °C. In order to determine the reactions occurring near these peaks, the Ni-Zr mixtures were heated to 600 °C, 950 °C, 1030 °C, 1080 °C, 1160 °C and 1200 °C, respectively, and then cooled down. XRD patterns for the DSC products of Ni-Zr mixtures quenched at different temperatures are shown in Figure 4. When the Ni-Zr mixture was heated to 600 °C, only the original reactants Ni and Zr were found in the quenched product, and no obvious reaction occurred (see Figure 4). When the Ni-Zr mixture was heated to 950 °C, the diffraction peak intensity of Ni and Zr in the quenched product was obviously weakened. At this time, NiZr, Ni_10_Zr_7_ and Ni_5_Zr were generated, which indicated that there was a solid-state reaction between Ni and Zr, resulting in a wide exothermic peak at 878 °C. When the Ni-Zr mixture was heated to 1030 °C, the content of Ni_10_Zr_7_ increased significantly, which corresponded to the exothermic peak at 1030 °C (see Figure 4). As the temperature was raised to 1080 °C, the Ni_11_Zr_9_ phase appeared, and the content of unreacted Ni and Zr decreased significantly. The production of Ni_11_Zr_9_ led to the presence of an exothermic peak at 1074 °C. As the temperature was raised to 1160 °C, Ni_10_Zr_7_ disappeared, and there was a large amount of NiZr and a small amount of Ni_11_Zr_9_ in the product. Following the Ni-Zr binary phase diagram [23], Ni_10_Zr_7_ and Ni will form a eutectic liquid at 1150 °C, which exactly corresponds to the endothermic peak at 1146 °C in the DSC curve. When the Ni-Zr mixture was heated to 1200 °C, the product mainly consisted of NiZr, Ni_11_Zr_9_ and a small amount of NiZr_2_, in which the content of Ni_11_Zr_9_ phase increased obviously. According to the Ni-Zr binary phase diagram [23], NiZr and Ni will form eutectic liquid phase at 1170 °C. Therefore, it can be deduced that the Ni-Zr eutectic liquid will form after the temperature is gradually raised to 1170 °C, which leads to the endothermic peak at 1181 °C. Subsequently, when the mixture was heated to 1200 °C and then cooled down, Ni_11_Zr_9_ and NiZr_2_ eventually crystallized from the Ni-Zr eutectic liquid. 

**Figure 5:**
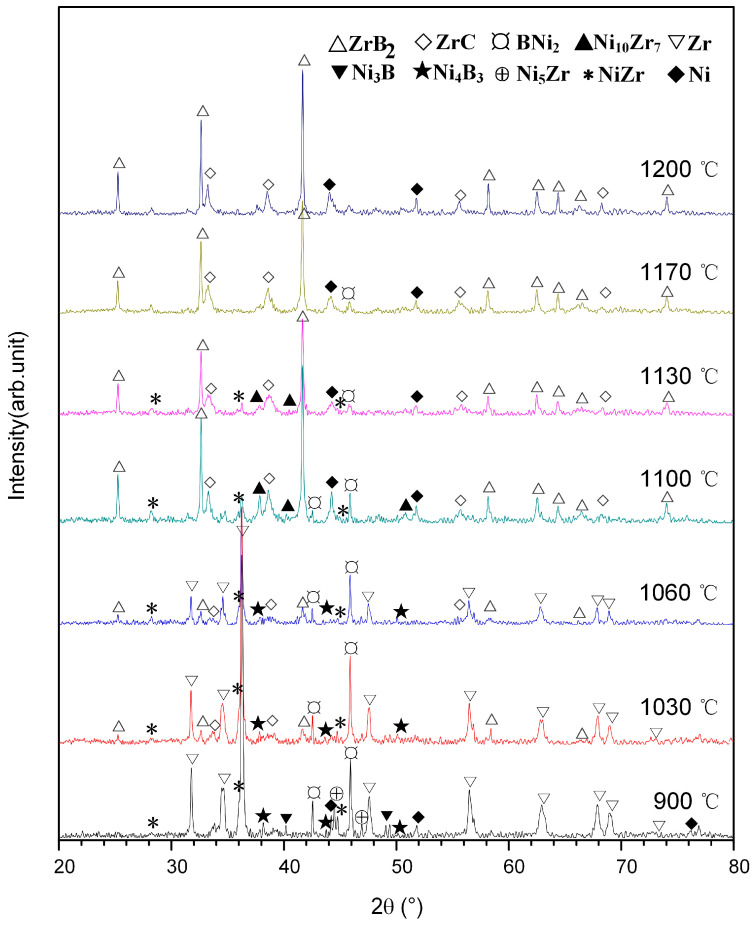
XRD patterns for the DSC products of 30 wt.% Ni-Zr-B_4_C mixtures quenched at different temperatures.

Figure 1d shows the DSC curve of the 30 wt.% Ni-Zr-B_4_C mixture heated to 1200 °C. As shown, two exothermic peaks were observed at 851 °C and 1088 °C, and two endothermic peaks were observed at 1025 °C and 1159 °C, respectively. In order to make clear the reactions occurring during the heating process, DSC interrupted experiments were carried out for the Ni-Zr-B_4_C mixtures at 900 °C, 1030 °C, 1060 °C, 1100 °C, 1130 °C, 1170 °C and 1200 °C, respectively, and then cooled down. The XRD patterns for the DSC products quenched at different temperatures are shown in Figure 5. When the Ni-Zr-B_4_C mixture was heated to 900 °C, a large amount of Ni_2_B and a small quantity of Ni_4_B_3_, NiZr and Ni_5_Zr were generated in the product, indicating that the wide exothermic peak near 851 °C corresponded to the formation of these Ni_x_Zr_y_ and Ni_x_B_y_ phases. This is also consistent with the previous analysis of Ni-Zr and Ni-B mixtures. When the Ni-Zr-B_4_C mixture was heated to 1030 °C, a very small amount of ZrB_2_ and ZrC appeared in the product, indicating that a small amount of Zr reacted with B_4_C at this time. As the temperature was raised to 1060 °C, the diffraction peak intensity of Ni_2_B and Ni_4_B_3_ decreased. According to the analysis of the Ni-B_4_C mixture, Ni_2_B and Ni_4_B_3_ can form the Ni-B eutectic liquid at 1018 °C, which corresponds to the endothermic peak at 1025 °C in the DSC curve of Ni-Zr-B_4_C. At the same time, the formation of the Ni-B liquid phase also promotes the contact and reaction between the reactants in the mixture, and the free C atomic can dissolve into the Ni-B liquid phase to form the Ni-B-C ternary liquid phase, which fully contacts with the surrounding Zr powder and B_4_C powder. As the temperature was raised to 1100 °C, a large amount of Ni, ZrB_2_ and ZrC were formed in the product, and a large exothermic peak appeared at 1088 °C in the DSC curve. It is speculated that part of the Zr powder directly reacted with B_4_C to form ZrB_2_ and ZrC, and part of the Zr powder dissolved into the surrounding Ni-B-C ternary liquid to form Ni-Zr-B-C quaternary liquid. When the concentration of [Zr], [B] and [C] atoms in the Ni-Zr-B-C liquid achieved the thermodynamic condition for the formation of ZrB_2_ and ZrC, ZrB_2_ and ZrC particles precipitated out of the saturated liquid. It is worth mentioning that a large amount of Ni_10_Zr_7_ also appeared in the product at 1100 °C, which was slightly different from the temperature at which Ni_10_Zr_7_ appeared in large quantities in the Ni-Zr mixture (1030 °C), which may be due to the influence of the addition of B_4_C in the Ni-Zr-B_4_C mixture. When the Ni-Zr-B_4_C mixture was heated to 1130 °C, the product was mainly composed of a large amount of ZrB_2_, ZrC and a small amount of Ni_10_Zr_7_ and Ni_2_B. When the Ni-Zr-B_4_C mixture was heated to 1170 °C, the product consisted of ZrB_2_, ZrC, Ni and a small amount of Ni_2_B. As the temperature was raised to 1200 °C, the product consisted of ZrB_2_, ZrC and Ni, indicating that the reaction of the system had tended to be complete. When the temperature rose from 1130 °C to 1170 °C, the content of Ni_10_Zr_7_ decreased rapidly, which was consistent with the results in the previously studied Ni-Zr mixture. When the temperature reached 1150 °C, Ni_10_Zr_7_ and Ni could form a Ni-Zr eutectic liquid phase, corresponding to the thermal absorption peak at 1159 °C in the DSC curve of the Ni-Zr-B_4_C mixture. The formation of Ni-Zr liquid phase promotes the contact and reaction between each component, which makes the reaction of the whole system fast and complete.

**Figure 6:**
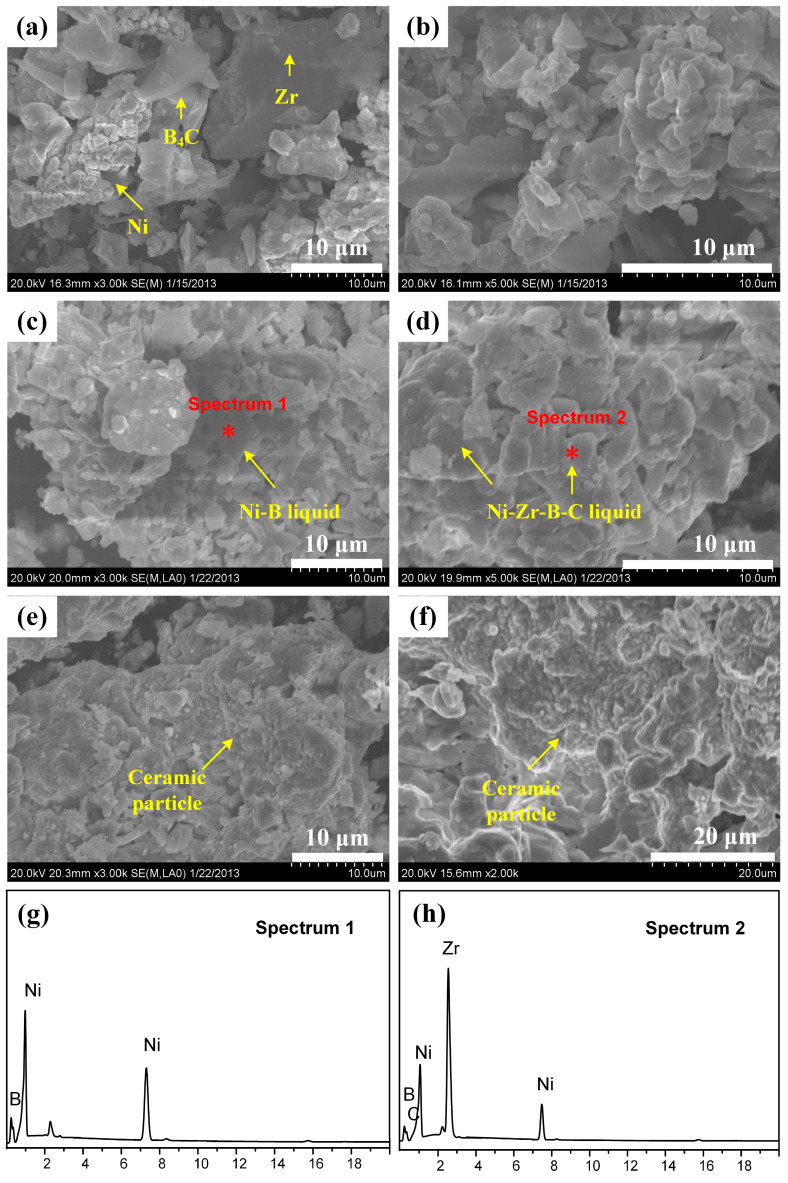
SEM micrographs for the DSC products of 30 wt.% Ni-Zr-B_4_C mixtures at (**a**) room temperature, quenched at (**b**) 900 °C; (**c**) 1060 °C; (**d**) 1100 °C; (**e**) 1170 °C; (**f**) 1200 °C; (**g**,**h**) the energy-dispersive spectrometry (EDS) spectra of (**c**,**d**).

In order to better illustrate the above viewpoints, microstructure analysis of DSC quenching products in the 30 wt.% Ni-Zr-B_4_C mixture at different temperatures was carried out. The SEM images are shown in Figure 6. It can be seen from Figure 6a that, at room temperature, the raw material mixed powder presented a loose and uniform microstructure. When the temperature was 900 °C, the Ni powder no longer presented a flower shape, but it became denser and bound more closely with the surrounding Zr powder and B_4_C powder, as shown in Figure 6b. Some Ni_x_B_y_ compounds formed around it by energy spectrum analysis. When the temperature rose to 1060 °C, the formation of a liquid phase was observed (see Figure 6c). Combining the EDS-point scanning spectrum (see Figure 6g) with the SEM image, point 1 was rich in Ni and B and thus mainly contained the Ni-B liquid phase. When the temperature rose to 1100 °C, the EDS-point scanning spectrum (see Figure 6h) of point 2 in Figure 6d contained Zr, Ni, B and C and, thus, possibly mainly contained the Ni-Zr-B-C liquid phases. When the temperature was further increased to 1170 °C, a large amount of liquid phase was formed, and a small number of ceramic particles were precipitated out of the liquid phase (see Figure 6e). When the temperature was increased to 1200 °C, a large number of ceramic particles formed in the product, as shown in Figure 6f. These results indicate that the microstructure evolution of DSC-quenched products is consistent with the previously inferred reaction mechanism analysis.

## 4. Conclusions

Based on DSC and XRD analysis of Zr-B_4_C, Ni-B_4_C, Ni-Zr and 30 wt.% Ni-Zr-B_4_C mixtures, the reaction mechanism in 30 wt.% Ni-Zr-B_4_C mixture under DSC conditions is proposed as follows: (i) Firstly, some intermetallic Ni_x_B_y_ (mainly Ni_2_B and Ni_4_B_3_) and Ni_x_Zr_y_ (mainly NiZr and Ni_5_Zr) formed via solid-state diffusion reactions of Ni, B_4_C and Zr at about 851 °C. (ii) Then, Ni_2_B and Ni_4_B_3_ formed a Ni-B eutectic liquid at about 1025 °C, and the free C atoms dissolved into the Ni-B liquid to form a Ni-B-C ternary liquid. When the mixture was heated to about 1088 °C, part of the Zr powder directly reacted with B_4_C through a solid-state diffusion reaction, and part of the Zr powder dissolved into the surrounding Ni-B-C ternary liquid to form Ni-Zr-B-C quaternary liquid. (iii) Finally, when the concentration of [Zr], [B] and [C] in the liquid attained a certain value, ZrB_2_ and ZrC formed and precipitated out of the saturated liquid.

## Data Availability

Not applicable.
